# Impact of tobacco smoking on estimated telomere shortening and epigenetic age acceleration among human blood immune cell types

**DOI:** 10.1186/s13148-026-02187-w

**Published:** 2026-06-30

**Authors:** Xuting Wang, Michelle R. Campbell, Suzanne N. Martos, Jian-Liang Li, Douglas A. Bell

**Affiliations:** 1 Immunity, Inflammation and Disease Laboratory, National Institute of Environmental Health Sciences, National Institutes of Health, Research Triangle Park, NC 27709 USA; 2 Biostatistics and Computational Biology Branch, National Institute of Environmental Health Sciences, National Institutes of Health, Research Triangle Park, NC 27709 USA

## Abstract

**Background:**

Tobacco smoke exposure has been shown to dramatically alter the methylome of whole blood DNA, and we have previously observed that effects are very different among blood cell types. In the present work, we compare smoking effects on DNA methylation-based telomere length (DNAmTL) shortening, age acceleration (DNAmAA) of 6 DNAmAge clocks (Horvath, Hannum, Skin_Blood, PhenoAge, FitAge, GrimAge2), aging pace (DunedinPACE), and Stochastic Epigenetic Mitotic Timer of Cancer (stemTOC) in six major immune cell types isolated from the whole blood samples of the same individuals from 74 nonsmokers and 69 smokers.

**Results:**

Telomere shortening in all cell types was significantly associated with smoking status, with smokers displaying greater telomere shortening. Examining the difference in DNAmTL shortening between smokers and nonsmokers across cell types, CD8+ T cells had the greatest DNAmTL shortening, while CD15+ granulocytes had the least shortening. Among six DNAmAge models tested, the strongest association between DNAmAge Acceleration (DNAmAA) and smoking status was observed for the GrimAge2 model, followed by the FitAge model and then the PhenoAge model, with smokers displaying greater age acceleration. For the GrimAge2 and FitAge models, as expected, smokers revealed greater age acceleration in whole blood samples. Across all cell types, the effect size in epigenetic age acceleration associated with smoking was significantly correlated with smoking effect as measured by mean *AHRR* cg05575921 demethylation values, with myeloid cell types showing the greatest effect and T cells least. However, the Hannum, Horvath, and Skin_Blood models (which were trained only by chronological age) were generally not significantly associated with smoking status in isolated cell types. We observed the DunedinPACE model was significantly associated with smoking status in all cell types and whole blood samples. Smokers had significantly higher aging pace in all cell types relative to nonsmokers. The stemTOC was not significantly associated with smoking status in isolated cell types.

**Conclusions:**

The present study demonstrates that tobacco smoking is significantly associated with methylation-based measures of telomere length shortening, biological age acceleration, and aging pace in six major immune cell types and whole blood. The effect of smoking on these outcomes differs among cell types, suggesting shifts in cell composition may play an important role in human aging as measured by DNA methylation models and epigenetic aging may play a role in smoking-related diseases.

**Supplementary Information:**

The online version contains supplementary material available at https://doi.org/10.1186/s13148-026-02187-w.

## Background

Tobacco smoking is associated with cancers, respiratory and cardiovascular diseases, and many adverse health outcomes. Tobacco smoke exposure has been shown to dramatically alter the methylome of whole blood DNA isolated from both adults and neonates [1, 2]. Su et al. [3] and Wang et al. [4] demonstrated that smoking-associated DNA methylation alterations differ among six major immune cell types (CD14+ monocytes, CD15+ granulocytes, CD19+ B cells, CD56+ natural killer cells, CD4+ T cells, and CD8+ T cells) in human blood and that some effects are highly cell-type specific. Altered methylation at CG dinucleotides (CpG sites) has been firmly established as biomarkers of chronological aging, which forms the basis for epigenetic aging models [5]. A person’s methylation-based biological age may be either older or younger than their chronological age, reflecting his/her aging and health status [6]. Research publications from the Horvath group [7] and Hannum et al. [8] have established that DNA methylation status in blood and other tissues can be used to identify individuals who appear to exhibit a reduced or accelerated estimate of methylation-based biological age. The Horvath model (353 CpGs) was developed using methylation data from multiple tissues [7] while the Hannum model (71 CpGs) was based primarily on whole blood DNA [8]. Follow-up studies reported that methylation-based age acceleration (DNAmAA) was strongly correlated with numerous adverse health outcomes such as cardiovascular disease, all-cause mortality [9] and time to death. Similarly, methylation levels at CpG dinucleotides have been used to approximate various forms of biological aging. Levine et al. developed ‘PhenoAge’ comprising 513 CpGs as biomarkers of a ‘phenotypic age’, which may better reflect one’s health condition than chronological age [10]. Lu et al. also proposed ‘GrimAge’ and “GrimAge version 2” incorporating 1030 CpGs as biomarkers of smoking pack-years and seven morbidity-associated plasma proteins [11, 12]. McGreevy et al. [13] developed the epigenetic clock FitAge to incorporate physical fitness parameters and integrated it with GrimAge. These measures have been shown to correlate with changes in molecular signs of decline and can provide further insights into the effect of lifestyle on the aging process. Belsky et al. [14] developed DunedinPACE using DNAm of 173 CpGs measured at a single timepoint to estimate the collective, time-dependent changes in various biological systems; it is proposed to measure the rate, or pace, of aging. The values produced by the DunedinPACE metric are scaled to have a mean of 1, representing the average change that occurs over one year. Recently, Zhu et al. [15] constructed an improved pan-tissue DNAm counter of total mitotic age called stemTOC (Stochastic Epigenetic Mitotic Timer of Cancer), which utilized 371 CpGs to detect mitotic-age increases (thought to be a major determinant of cancer-risk) in preneoplastic lesions and tumors relative to healthy tissue.

Epigenetic age acceleration represents the relative difference between individual’s chronological age and epigenetic age, where positive values of epigenetic age acceleration indicate faster biological aging. A study found smoking accelerated aging of airway cells and lung tissue, and quitting smoking reduced epigenetic aging in airway cells [16]. Of note, the GrimAge models [11, 12] explicitly include the methylation-based smoking biomarker CpG cg05575921 in the *AHRR* gene and this contributes to prediction of time-to-death.

Cellular telomeres are complex DNA–protein structures located at the end of eukaryotic chromosomes, and telomere length has been shown to shorten as a result of cell replication [17]. Molecular assays of telomere length in blood DNA in many large population studies reveal an inverse correlation between chronological age and telomere length [18]. The NIH funded Genotype-Tissue Expression (GTEx) project has reported that telomere length can vary significantly across many different human tissue types [19] and that tissue cell division, age, race and polygenic genetic risk score all contribute to variation in telomere length. In that study, whole blood DNA telomere length displayed the strongest association with age among tissues. However, different blood cell types were not evaluated. Smoking history has been associated with blood DNA-based molecular measurements of telomere length [20]. Comparing telomere shortening in ever smokers with that in never smokers may reveal mechanisms connecting smoking with aging-related disease [20]. Recently the Horvath group has developed and validated a DNA methylation-based model for estimating telomere length, referred to as DNAmTL, against a large set of population samples with previous biological determination of telomere length [21]. We used this technique to compare estimates of telomere length among immune cell types.

In the present work, we used currently available methylation-based models to analyze telomere length shortening, age acceleration, aging pace, and stemTOC in six major immune cell types isolated from whole blood samples of the same individuals, a group of 74 nonsmokers and 69 smokers (as in Wang et al. [4]). The objective was to compare the impact of tobacco smoke exposure on estimated telomere shortening and epigenetic clock acceleration (DNAmAA) across immune cell types in human blood, as well as examine how shifts of naïve and memory cells affect these clocks. For each methylation-based method we observed that outcomes differ across cell types and the effect of smoking on these outcomes differs among cell types.

## Methods

### Study population, peripheral blood leukocyte subtype isolation, and methylation analysis

The study description and demographics of the Epigenetic Biomarkers of Tobacco Smoke Exposure project have been described previously [4] and are summarized in Table S1. In addition, peripheral leukocyte cell type isolation and methylation analysis were also described in our previous report [4]. The raw IDAT files have been deposited in Gene Expression Omnibus (GEO) database under the accession numbers GSE224807 and GSE318669 (new data). Smoking was coded as current smoking (yes/no), detailed information on smoking exposure is available from the authors upon request. Comparisons between smoking and model outcomes used the average methylation level of *AHRR* CpG cg05575921 from our previous report [4].

### Cell type deconvolution

Each isolated cell-type sample may contain small quantities of contaminants (i.e. other cell types), and we can estimate cell-type composition using established DNA methylation-based cell type deconvolution models. For each sample, we estimated the proportions of six immune cell subtypes (CD14+ monocytes, CD15+ granulocytes, CD19+ B cells, CD56+ natural killer cells, CD4+ T cells, and CD8+ T cells) using the method of Houseman et al. [22]. Similarly, twelve leukocyte subsets were estimated using the method of Salas et al. [23]. In addition to leukocyte subsets from both the myeloid (i.e. neutrophil, basophil, eosinophil, and monocyte) and lymphoid lineages (i.e. B cells, CD4+ T and CD8+ T cells, natural killer and T regulatory cells), this model distinguishes between naïve and memory states for B and T cells. The cell type and memory state proportions were used in analyses to adjust for confounding effects and to explore whether associations were driven by shifts from naïve to memory among B and T cell types.

### Calculation of DNA methylation-based telomere length and epigenetic ages

We used Dr. Horvath’s online Methylation Age Calculator (https://dnamage.clockfoundation.org/) to calculate DNAmTL and six DNAm Age clocks, including Horvath, Hannum, Skin_Blood, PhenoAge, GrimAge2, and FitAge. The Bioconductor ‘minfi’ R package was used to process the DNA methylation data. The raw data IDAT files were read into R using the ‘read.metharray.exp’ function, and the ‘combineArrays’ function was used to combine different generations of Illumina Infinium DNA methylation arrays (450k and EPIC). Then the ‘preprocessNoob’ function was used to perform ‘noob’ (normal-exponential using out-of-band probes) normalization. After missing data points were filled up with “NA” (as recommended by the Age Calculator), the matrix of methylation levels was uploaded into the online calculator. Then the epigenetic clock indices were calculated with the “advanced analysis” option and the “normalize data” option. We additionally calculated the principal component (PC) versions of Horvath, Skin_Blood, Hannum, PhenoAge, GrimAge, and DNAmTL as describe in [24] to assess possible noise reduction benefit.

### Calculation of DunedinPACE

The DunedinPACE epigenetic clock was calculated using the methylAge function within the ENmix R package [25].

### Calculation of stemTOC

The raw idat files were processed with minfi preprocessNoob function, followed by BMIQ Normalization. Then stemTOC values were calculated using the R package EpiMitClocks [15].

### Statistical analysis

All statistical analyses were conducted using the open-source R program (https://www.r-project.org/, version 4.3.3). For whole blood samples and each cell type, individuals’ DNAmTL shortening or DNAmAA values were calculated using the residuals of a linear regression of DNAmTL or DNAmAge against chronological age [26]. The association between DNAmAA and smoking status was evaluated using multiple linear regression models, including: (1) “unadjusted” model, which is “lm(DNAmAA ~ Smoking_status)”, (2) “adj” model, which is “ lm(DNAmAA ~ Smoking_status + sex + race + BMI + age)”, (3) “adj_cells6” model, which is “ lm(DNAmAA ~ Smoking_status + sex + race + BMI + age + predicted 6 cell-types)”, and (4) “adj_cells12” model, which is “lm(DNAmAA ~ Smoking_status + sex + race + BMI + age + predicted 12 cell-types)”. Models 2 through 4 were used to assess the effect of potential confounding factors including age, race, sex and cell-type composition. For DNAmTL analysis, DNAmTL shortening replaces DNAmAA in the above models. For stemTOC analysis, stemTOC replaces DNAmAA in the above models.

## Results

### Demographics

Six major cell types were isolated from whole blood (WB) from volunteer adult smokers (SM, *n* = 69) and nonsmokers (NS, *n* = 74) living in the Raleigh, Durham and Chapel Hill, North Carolina region. DNA methylation profiles from the cell types and whole blood samples were determined using Illumina 450k or EPIC arrays (as reported in [4]). Nonsmokers were younger than smokers (39.0 ± 1.2 yrs versus 43.2 ± 1.1 yrs; p = 0.01) and had a greater mean BMI (30.2 ± 0.9 versus 28.0 ± 0.6; p = 0.036). See Table S1 for the summary of demographic variables.

### DNAmTL varies across blood cell types and is altered by smoking

**Fig. 1:**
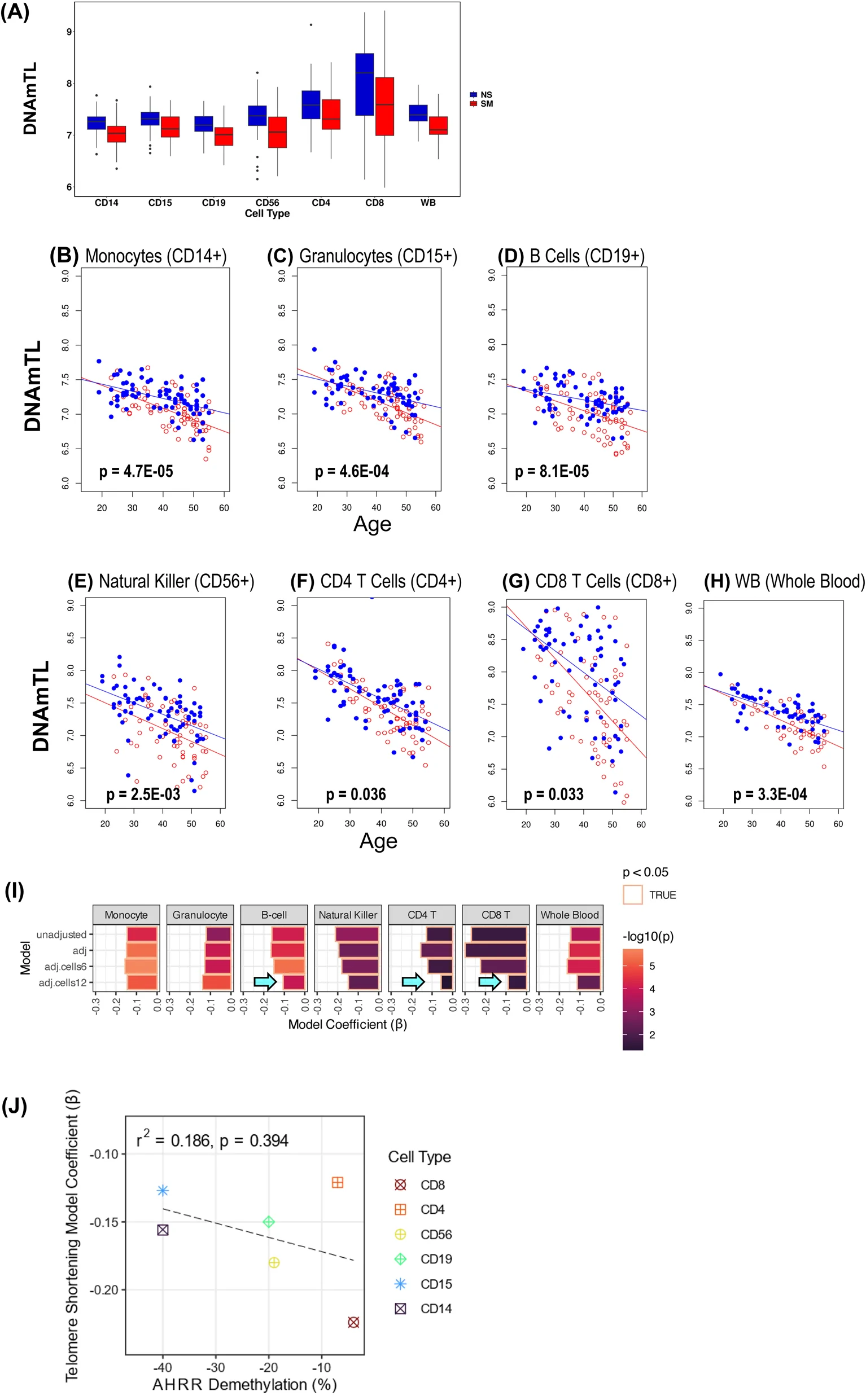
Smoking effect on DNAmTL and its shortening in blood cell types. **A** Boxplot of DNAmTL in cell types and matched whole blood from nonsmokers (NS) and smokers (SM). **B**–**H** Scatter plots display DNAmTL vs. Age. The red circles represent smokers, and the blue dots represent nonsmokers. The trend lines represent linear regression of DNAmTL on age for nonsmokers and smokers. In each plot, the *p*-value is the significance of the difference between the two trend lines for nonsmokers and smokers (blue = nonsmokers; red = smokers). **I** Impact of cell type adjustment on the coefficient (β, or beta) of association tests between DNAmTL shortening and smoking status. **J** Smoking-associated DNAmTL shortening effect size (model adj.cells6 coefficient β) in relation to smoking biomarker (AHRR) demethylation

Using DNA methylation profiles, we estimated telomere length (DNAmTL) for each sample using a method described by Lu et al. [21] based on methylation levels at 140 CpGs that were previously associated with laboratory measurements of telomere length. Figure 1A shows the distribution of DNA methylation-based estimation of DNAmTL across cell types and whole blood for the nonsmokers and smokers in this study. We observed that smokers have shorter average DNAmTL in each cell type and whole blood compared to nonsmokers. Additionally, CD4 T and CD8 T cells exhibited longer average DNAmTLs relative to other cell types, in both nonsmokers and smokers.

From the scatter plots of DNAmTL versus age (Fig. 1B–H), we observed that older subjects in both smokers and nonsmokers had shorter DNAmTL in each cell type and whole blood. Furthermore, the trend lines show that smokers have significantly shorter DNAmTL than nonsmokers, even accounting for chronological age.

We further used linear regression analysis to examine the association between smoking status and DNAmTL shortening in cell types or whole blood, with adjustment for potential confounding factors such as age, race, sex, BMI and cell composition. For each isolated cell-type sample, small quantities of other cell types may be present, and we can estimate proportions using cell deconvolution models [22, 23]. The effect of other cell types on the association test can be adjusted (or regressed out) using multiple linear regression models. Using the six cell-type adjusted model (adj.cells6) and the twelve cell-type adjusted model (adj.cells12), we examined the effect of smoking on the shortening of DNAmTL in each cell type, which was defined using residuals from the linear regression of the DNAmTL on chronological age.

In Fig. 1I a comparison of adjusted models is displayed, including a six cell-type model (adj.cells6) and a twelve cell-type model (adj.cells12). The bar graph in Fig. 1I shows the effect size coefficients (or slopes) of different regression models. We observed that smoking status was significantly associated with DNAmTL shortening in all cell types and in matched whole blood before (unadjusted) and after adjustment for potential demographic confounders (adj) and cell composition (adj.cells6 and adj.cells12), with smokers having significant DNAmTL shortening relative to nonsmokers. As further verification, methylation data from an additional group of whole blood samples from a larger population (n = 253) of smokers and nonsmokers from our previous study of Su et al. [3] (see Table S2 column “Su et al. Whole Blood”) was analyzed. This analysis (Table S2) displayed a highly significant DNAmTL shortening relative to smoking, consistent with the result observed in the present set of samples. CD14+ monocytes displayed the strongest significant association between smoking and DNAmTL shortening (i.e. smallest *p*-value) while the largest magnitude of DNAmTL shortening was in CD8 T cells (Fig. 1I; Table S2). Notably, the effect size of smoking on DNAmTL shortening with the 12 cell-type adjustment model was greatly attenuated in B and T lymphocytes (indicated with cyan arrows on Fig. 1I). The detailed statistical results are in Table S2.

Figure 1J displays the correlation between smoking-associated DNAmTL shortening effect size (adj.cells6 model coefficient, beta) and demethylation at the smoking biomarker *AHRR* cg05575921, with a trend line showing a lack of association with smoking effect size across cell types (r^2^ = 0.186 *p* = 0.394). Myeloid cells showed the largest smoking-associated changes in *AHRR* methylation but relatively low smoking-associated DNAmTL shortening. In contrast, CD8 T cells showed minimal smoking-associated *AHRR* methylation changes, but substantial smoking-associated DNAmTL shortening.

### Smoking-associated epigenetic age acceleration among immune cells

The association of DNAmAA (defined as residuals of a linear regression of DNAmAge against chronological age [26]) with smoking status was evaluated in the same way described as in the DNAmTL analysis using multiple linear regression models (unadjusted, adj, adj.cells6, and adj.cells12).

**Fig. 2:**
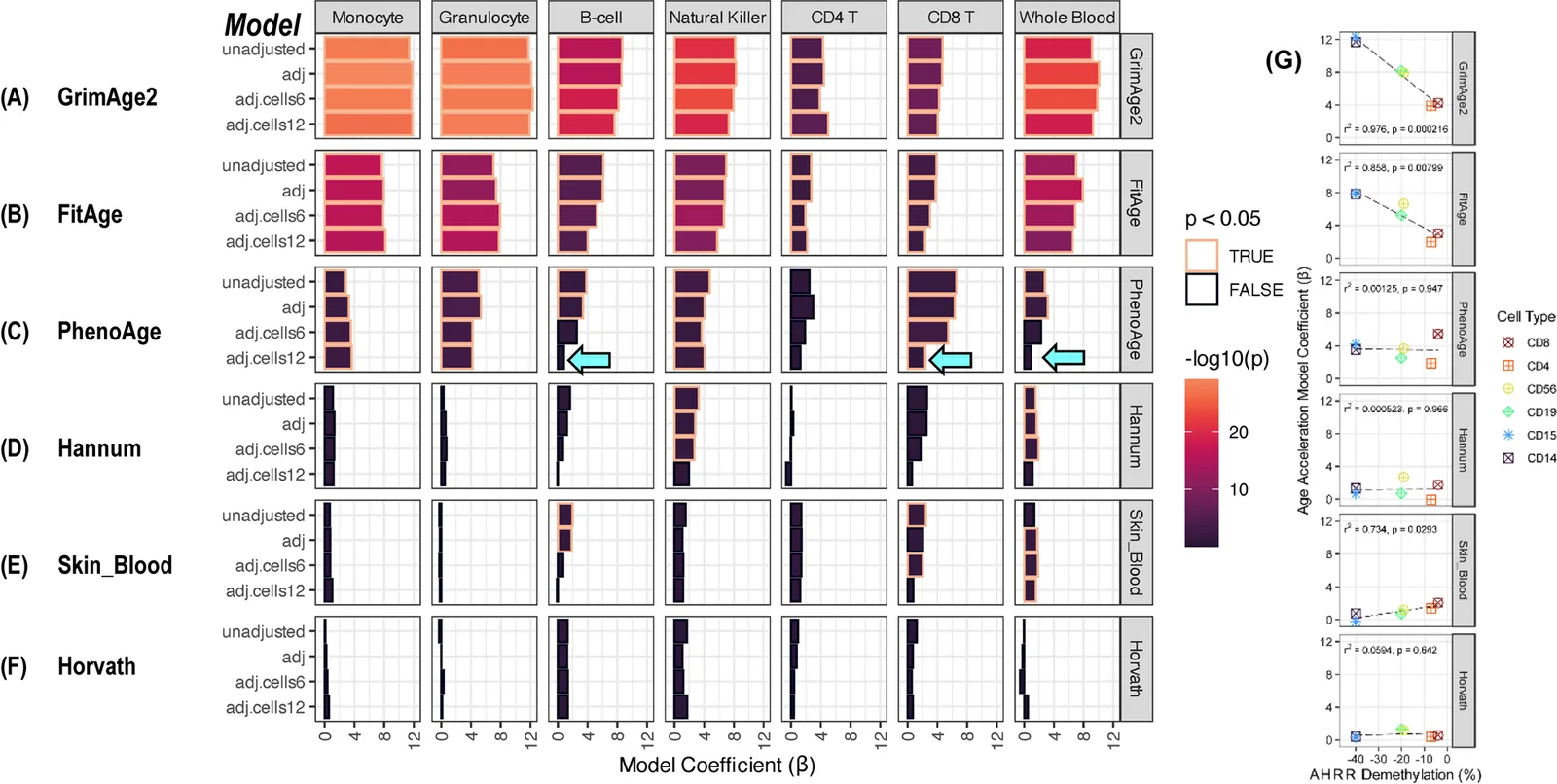
Smoking effect on DNAmAA in blood cell types. **A**–**F** Smoking-associated altered DNAmAA assessed with six methods in isolated cell types and matched whole blood. **G** Smoking-associated DNAmAA effect size (model adj.cells6 coefficient, β) in relation to the smoking biomarker (*AHRR*) demethylation in each cell type

Figure 2 (A-F) displays a graphical summary of the association results for each model among cell types and matched whole blood. Table S3 provides the *p*-values (unadjusted and adjusted) and ΔDNAmAA for each cell type, matched whole blood, and Su et al. whole blood. Table S3 also lists a measure of smoking effect size represented by the % demethylation at the *AHRR* cg05575921 CpG by cell type. A higher negative demethylation value indicates a larger smoking effect. Among all DNAmAge models, the strongest association (smallest *p*-value) with smoking status was observed for the GrimAge2 model (Fig. 2A), with smoking significantly associated with DNAmAA in all cell types and in two separate whole blood sample sets (Table S3, matched whole blood and Su et al. whole blood). It is not surprising that a strong association was found by the GrimAge2 model as this model explicitly incorporates ten DNAm-based surrogate markers for smoking pack-years [11, 12]. It is notable that the GrimAge2 model was highly significant even in T cell subsets that show modest smoking effects based on the *AHRR* smoking biomarker (Fig. 2G).

The results for the FitAge model (Fig. 2B; Table S3), which shares parameters with GrimAge2, were similar. Smoking status was significantly associated with DNAmAA in both whole blood sample sets, as expected, and in the isolated cell type samples. Among cell types the FitAge DNAmAA was correlated with *AHRR* cg05575921 demethylation. Adjustment for estimated cell-type proportions did not strongly affect FitAge results.

The PhenoAge model was trained based on nine different clinical measures of phenotypic biomarkers of aging observed in blood such as C-reactive protein (CRP, a biomarker of inflammation) and immune cell-type counts [10]. Using the PhenoAge model, smoking was significantly associated with DNAmAA in both sets of whole blood samples and in all cell types except for the CD4 T cells before (unadjusted) and after adjusting for sex, race, BMI and age (adj model). However, further adjustment for six (adj.cells6) or twelve (adj.cells12) estimated cell-type percentages resulted in attenuation of the smoking-associated DNAmAA effect size values in the B cells, CD8 T, and whole blood samples (Fig. 2C cyan arrows; Table S3). Smoking-associated DNAmAA remained significant in CD8 T cells after cell composition adjustment but the effect was somewhat reduced after six cell-type adjustment and substantially reduced after twelve cell-type adjustment. PhenoAge DNAmAA was no longer significantly associated with smoking status in whole blood data sets after cell composition adjustment.

The Hannum DNAmAA (Fig. 2D; Table S3) was associated with smoking status only in the CD56 fraction and in the matched whole blood set. Adjusting for cell composition by twelve but not six cell types attenuated the effect. The Skin_Blood DNAmAA was associated with smoking status in the B cell fraction only before cell-type percentage adjustment, while CD8 T cell association became more significant after adjusting for six cell types (Fig. 2E; Table S3). Interestingly, the fraction-matched whole blood did not show a smoking status association before adjustment, but was significant after all three adjustments. Horvath DNAmAA was not associated with smoking status in any cell fraction or whole blood (Fig. 2F; Table S3).

Figure 2G displays the relationship between the effect size (i.e. beta coefficient from the adj.cells6 model) and the *AHRR* cg05575921 demethylation value for each cell type (i.e. mean methylation of smokers minus that of nonsmokers). GrimAge2 effect size was strongly negatively associated with *AHRR* cg05575921 demethylation values (r^2^ = 0.976, *p* = 0.000216), with myeloid cell types showing the greatest effect and T cells the least. Adjustment for estimated cell-type proportions did not greatly affect GrimAge2 (Fig. 2A; Table S3). Similar to the GrimAge2 result, the effect size values of FitAge were strongly negatively associated with cell-type *AHRR* cg05575921 demethylation values (r^2^ = 0.858, p = 0.00799), with myeloid cell types showing the greatest effects for DNAmAA and T cells the least. PhenoAge, Hannum, and Horvath cell-type DNAmAA effect sizes were not associated with smoking-related cell-type *AHRR* demethylation (Fig. 2G). Skin_Blood DNAmAA effect size among cell types displayed a modest relationship to smoking (r^2^ = 0.734, *p* = 0.0293) (Fig. 2G).

A recent report has suggested that a principal component (PC) version of the DNAmTL and DNAmAA models [24] could reduce technical variation and bolster reliability. We calculated the PC scores and ran through each of the models described as above; the overall outcome (see Tables S5 and S6) aligns well with that from non-PC scores shown above.

### Smoking association with aging pace among cell types and whole blood

**Fig. 3:**
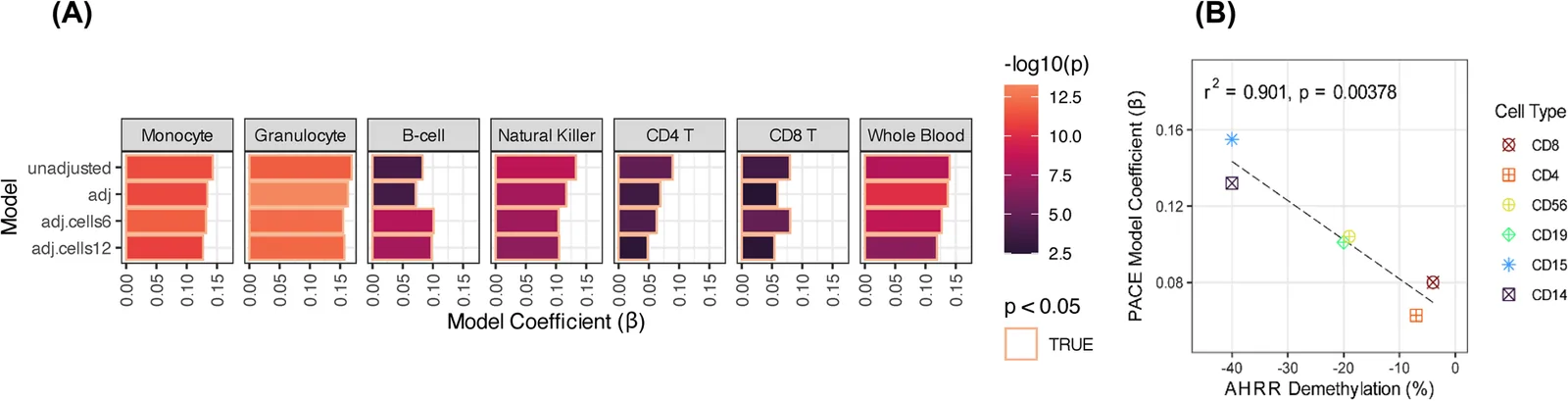
Smoking effect on DunedinPace in blood cell types. **A** Smoking effect on PACE assessed with adjusted models in isolated cell types and matched whole blood. **B** PACE effect size (model adj.cells6 coefficient, β) in relation to smoking biomarker (*AHRR*) demethylation in each cell type

Using the DunedinPACE model [14], we quantified pace of aging for all samples. We also examined the association between DunedinPACE value and smoking status, with adjustment for sex, race, BMI, age and predicted cell types. We observed smokers had significantly higher DunedinPACE aging values relative to nonsmokers in all cell types and whole blood (Fig. 3A; Table S4). Adjustment for estimated cell types also generally attenuated the smoking effect and significance, although B cells were an exception. Compared to adjusting for demographic factors (adj), CD8 T cells had a larger effect size in the six cell-type adjustment model, but this effect size was reduced after adjusting for twelve cell types.

The PACE effect size (i.e. beta coefficient of model adj.cells6) in each cell type was negatively correlated with the *AHRR* cg05575921 demethylation values (Fig. 3B), with myeloid cell types showing the greatest smoking effects for PACE effect and T cells the least.

### Association of smoking with stemTOC among cell types and whole blood

Zhu et al. [15] observed a significant positive correlation of stemTOC with smoking status in a dataset of 790 buccal swabs from healthy women all aged 53 at sample draw and another dataset of 204 normal lung tissue samples. Using the stemTOC algorithm, we calculated this mitotic age for all samples and examined the association between stemTOC values and smoking status, with adjustment for sex, race, BMI, age and predicted cell types. The results are listed in Table S7.

Without adjusting for sex, race, BMI, and age, we observed a significant positive correlation of stemTOC with smoking status in B cell, CD8T, and NK cells. But after adjusting sex, race, BMI, and age, these associations were no longer significant. For the whole blood samples, we observed a significant positive correlation of stemTOC with smoking status in the unadjusted model and the effect was attenuated in the adjusted models.

## Discussion

Epigenetic age acceleration and telomere shortening have been reported to be biomarkers of aging [7, 8, 10, 11, 21], associated with age-related disease [27–30] and potential predictors of chronic disease risk. It is not surprising that tobacco smoking, which affects numerous acute and chronic health outcomes, has been associated with DNAmAA and telomeres in many studies of human blood [10, 20, 31]. The present study provides support for these findings and reveals additional details as to immune cell-type specific impacts of tobacco smoking.

Telomere shortening in blood cells (DNAmTL) has been associated with age-related diseases and is highly heritable [32, 33], however, in contrast, cells with longer telomeres are predisposed to cancers [34]. Telomere shortening rate predicts a species lifespan in different tissues and cell types and reportedly outperforms Terminal Restriction Fragment (TRF)-based Leukocyte Telomere Length (LTL) in predicting mortality and time-to-heart disease, as well as other age-related conditions [35]. In blood cell types and whole blood samples we observe strong correlations between age and DNAmTL (Fig. 1B-H) and significantly shortened DNAmTL in smokers when plotted against age. However, telomere length and the shortening associated with smoking differed between T cells and other immune cell types. In nonsmokers, DNAmTL was significantly longer among isolated CD4 T and CD8 T cell samples (Fig. 1A) but displayed disparate smoking effects (Fig. 1F-G). In particular, the detected difference in DNAmTL between nonsmokers and smokers was quite large in CD8 T cells but relatively small for CD4 T cells. The effect of smoking, as measured by the methylation-based smoking biomarker (*AHRR* cg05575921), is relatively small in both T cell types [3, 4] compared to myeloid cells and whole blood. For CD8 T cells, the weak association between DNAmTL shortening with *AHRR* demethylation combined with the large variance among individuals, suggests other mechanistic causes are involved. For example, T cell proliferation related to chronic viral infections like cytomegalovirus may impact T cell methylation profiles [36] and could be affecting the measurement of DNAmTL [37]. In the regression models testing the smoking effect on DNAmTL (Fig. 1J), the attenuation of effect size (model coefficient) and statistical significance following adjustment for naïve versus memory CD8 T cells (Fig. 1J; Table S2) suggests there is a cell differentiation state shift that is associated with smoking, either directly or indirectly affecting proliferation history.

In a human population sample, the correlation between DNA methylation at thousands of CpG loci and age is striking and can be used to predict chronological age very accurately for most individuals. However, Horvath [7] reported that some individual’s methylation-based age estimates were older than their chronological age, thus the term “age acceleration” was coined. Many DNAmAge models estimating biological age have associated methylation-based aging values with a variety of adverse outcomes and diseases, however, the underlying biology of most of these correlations is not well understood. Regarding DNA methylation measurements in whole blood, it has been well known that cell-type composition shifts as the immune system ages [38, 39]. Specifically, as humans age, with exposure to pathogens, there is a shift from naïve to memory among T and B cells, an increase in exhausted and senescent cells [40], and changes in myeloid and erythroid cell counts. It is recognized that these cell type shifts correlate with methylation changes. We have previously reported changes in CD8 T cell subpopulations with smoking and a concomitant effect on T cell DNAmAA using the PhenoAge model [41]. The PhenoAge model, which was trained on numerous blood parameters, shows the impact of adjustment for naïve vs memory cells in T and B cell types and in whole blood (see adj.cells6 versus adj.cells12 results) (Fig. 2C, arrows).

To compare the smoking effect with model outcomes, we used the methylation level of *AHRR* CpG cg05575921, which is a sensitive and quantitative indicator of tobacco smoking exposure and is reportedly more reliable than self-reported smoking such as the number of cigarettes smoked per day [42, 43]. Comparing the effect of smoking exposure on DNAmAA models among all blood cell types, we observe that DNAmAA effect size for some models is correlated with smoking effect represented by demethylation at *AHRR* cg05575921 in that cell type. The GrimAge2 model specifically weights smoking exposure, and the *p*-values associated with DNAmAA due to smoking are similar in magnitude to the values obtained for smoking-related changes in methylation at known smoking-altered CpGs (*p*-values ranging from 10E-05 to 10E-28). Among cell types, GrimAge2 DNAmAA effect size and *AHRR* cg05575921 demethylation were strongly negatively correlated (r^2^ = 0.96, *p* = 6.67 × 10–^17^), indicating cell types displaying greater smoking effects show greater age acceleration. Similarly, FitAge model integrates GrimAge2 parameters and is trained on additional measures affected by smoking (forced expiratory volume in one second and maximal oxygen uptake). A smoking effect is also strongly observed in the DunedinPACE model, which was trained on longitudinal change in 19 biomarkers measuring integrity of the cardiovascular, metabolic, renal, hepatic, pulmonary, periodontal, and immune systems in a cohort of individuals of the same chronological age. This model appears more robust for detecting effects of environmental exposures (such as smoking) and survival bias, and notably more sensitive to anti-aging interventions [14]. Analysis of DNAmAA among cell types and blood using the Hannum, Skin_Blood and the original Horvath models (trained to estimate chronological age) provided relatively modest effect sizes and significance values relative to smoking exposure; this is consistent with other reports [44]. The StemTOC model estimates mitotic age in biological samples [15] but was generally not significantly associated with smoking in isolated cell types after adjustment. Whereas the previous stemTOC study examined the effect of smoking in a large sample of epithelial cells (buccal and lung) in older smokers, the ages examined here, and the size of the current study may be too small to detect the effect of smoking on StemTOC in blood cells.

## Strengths and limitations of the study

The cell-type specific analysis reveals that smoking impacts age-related changes in methylation outcomes differently among immune cell types in this population sample. This aging-related comparison across the major immune cell types has not been reported previously and reveals possible cell-type specific impacts on biological aging of the immune system. Recently developed principal component (PC) versions [24] of the traditional (non-PC) epigenetic clocks use PC adjustment to reduce technical variability in analyzed datasets. Comparing PC adjusted results with the non-PC (or traditional) epigenetic clocks, the overall outcomes align well (Tables S5-S6). A more detailed analysis of smoking behavior might reveal additional differences in outcome between heavy and light smoking. While the study provides clear support for the notion that smoking diminishes the immune system, the relatively limited sample size and age range (19–56 years of age) of the study group may reduce conclusions regarding the effects in older smokers. It seems possible that the effects we observed in this under 56-year-old population might be even greater in older smokers.

## Conclusion

The present study demonstrates that tobacco smoking is significantly associated with telomere length shortening measured by methylation (DNAmTL), biological age acceleration as measured by several DNAmAA models, and aging PACE in six major immune cell types and whole blood. The effect of smoking on these outcomes differs markedly among cell types. Shifts in cell composition are well known to occur with age, and smoking appears to exacerbate these changes. It will be of interest to determine the role that accelerated epigenetic aging has in the development of smoking-related diseases.

## Supplementary Information

Additional file 1

## Data Availability

The datasets generated and/or analyzed during the current study are available in GEO (GSE224807 and GSE318669). The R scripts are available from the corresponding author on request.

## Abbreviations

Adj: Linear regression model with adjustment for demographic factors (age, sex, race, and BMI)
Adj.cells6: Linear regression model with adjustment for six cell types and demographic factors
Adj.cells12: Linear regression model with adjustment for twelve cell types and demographic factors
BMI: Body mass index
BMIQ: Beta-mixture quantile
DNAmAge: DNA methylation-based age
DNAmAA: Epigenetic age acceleration
DNAmTL: DNA methylation-based telomere length
∆EAA: Difference in epigenetic age acceleration due to smoking
Gran: Granulocyte
Mono: Monocytes
NK: Natural killer
NIEHS: National Institute of Environmental Health Sciences
NIH: National Institutes of Health
stemTOC: Stochastic epigenetic mitotic timer of cancer
WB: Whole blood

## References

[1] Joehanes R, Just AC, Marioni RE, Pilling LC, Reynolds LM, Mandaviya PR, et al. Epigenetic signatures of cigarette smoking. Circ Cardiovasc Genet. 2016;9:436–47. 27651444 10.1161/CIRCGENETICS.116.001506 PMC5267325

[2] Joubert BR, Haberg SE, Nilsen RM, Wang X, Vollset SE, Murphy SK, et al. 450K epigenome-wide scan identifies differential DNA methylation in newborns related to maternal smoking during pregnancy. Environ Health Perspect. 2012;120:1425–31. 22851337 10.1289/ehp.1205412 PMC3491949

[3] Su D, Wang X, Campbell MR, Porter DK, Pittman GS, Bennett BD, et al. Distinct epigenetic effects of tobacco smoking in whole blood and among leukocyte subtypes. PLoS ONE. 2016;11:e0166486. 27935972 10.1371/journal.pone.0166486 PMC5147832

[4] Wang X, Campbell MR, Cho HY, Pittman GS, Martos SN, Bell DA. Epigenomic profiling of isolated blood cell types reveals highly specific B cell smoking signatures and links to disease risk. Clin Epigenetics. 2023;15:90. 37231515 10.1186/s13148-023-01507-8 PMC10211291

[5] Horvath S, Zhang Y, Langfelder P, Kahn RS, Boks MP, van Eijk K, et al. Aging effects on DNA methylation modules in human brain and blood tissue. Genome Biol. 2012;13:R97. 23034122 10.1186/gb-2012-13-10-r97 PMC4053733

[6] Jones MJ, Goodman SJ, Kobor MS. DNA methylation and healthy human aging. Aging Cell. 2015;14:924–32. 25913071 10.1111/acel.12349 PMC4693469

[7] Horvath S. DNA methylation age of human tissues and cell types. Genome Biol. 2013;14:R115. 24138928 10.1186/gb-2013-14-10-r115 PMC4015143

[8] Hannum G, Guinney J, Zhao L, Zhang L, Hughes G, Sadda S, et al. Genome-wide methylation profiles reveal quantitative views of human aging rates. Mol Cell. 2013;49:359–67. 23177740 10.1016/j.molcel.2012.10.016 PMC3780611

[9] Marioni RE, Shah S, McRae AF, Chen BH, Colicino E, Harris SE, et al. DNA methylation age of blood predicts all-cause mortality in later life. Genome Biol. 2015;16:25. 25633388 10.1186/s13059-015-0584-6 PMC4350614

[10] Levine ME, Lu AT, Quach A, Chen BH, Assimes TL, Bandinelli S, et al. An epigenetic biomarker of aging for lifespan and healthspan. Aging (Albany NY). 2018;10:573–91. 29676998 10.18632/aging.101414 PMC5940111

[11] Lu AT, Quach A, Wilson JG, Reiner AP, Aviv A, Raj K, et al. DNA methylation GrimAge strongly predicts lifespan and healthspan. Aging (Albany NY). 2019;11:303–27. 30669119 10.18632/aging.101684 PMC6366976

[12] Lu AT, Binder AM, Zhang J, Yan Q, Reiner AP, Cox SR, et al. DNA methylation GrimAge version 2. Aging. 2022;14:9484–549. 36516495 10.18632/aging.204434 PMC9792204

[13] McGreevy KM, Radak Z, Torma F, Jokai M, Lu AT, Belsky DW, et al. DNAmFitAge: biological age indicator incorporating physical fitness. Aging (Albany NY). 2023;15:3904–38. 36812475 10.18632/aging.204538 PMC10258016

[14] Belsky DW, Caspi A, Corcoran DL, Sugden K, Poulton R, Arseneault L, et al. DunedinPACE, a DNA methylation biomarker of the pace of aging. Elife. 2022;11:e73420. 35029144 10.7554/eLife.73420 PMC8853656

[15] Zhu T, Tong H, Du Z, Beck S, Teschendorff AE. An improved epigenetic counter to track mitotic age in normal and precancerous tissues. Nat Commun. 2024;15:4211. 38760334 10.1038/s41467-024-48649-8 PMC11101651

[16] Wu X, Huang Q, Javed R, Zhong J, Gao H, Liang H. Effect of tobacco smoking on the epigenetic age of human respiratory organs. Clin Epigenetics. 2019;11:183. 31801625 10.1186/s13148-019-0777-z PMC6894291

[17] Frenck RW Jr, Blackburn EH, Shannon KM. The rate of telomere sequence loss in human leukocytes varies with age. Proc Natl Acad Sci U S A. 1998;95:5607–10. 9576930 10.1073/pnas.95.10.5607 PMC20425

[18] Muezzinler A, Zaineddin AK, Brenner H. A systematic review of leukocyte telomere length and age in adults. Ageing Res Rev. 2013;12:509–19. 23333817 10.1016/j.arr.2013.01.003

[19] Demanelis K, Jasmine F, Chen LS, Chernoff M, Tong L, Delgado D, et al. Determinants of telomere length across human tissues. Science. 2020;369:eaaz6876. 32913074 10.1126/science.aaz6876 PMC8108546

[20] Astuti Y, Wardhana A, Watkins J, Wulaningsih W, Network PR. Cigarette smoking and telomere length: A systematic review of 84 studies and meta-analysis. Environ Res. 2017;158:480–9. 28704792 10.1016/j.envres.2017.06.038 PMC5562268

[21] Lu AT, Seeboth A, Tsai PC, Sun D, Quach A, Reiner AP, et al. DNA methylation-based estimator of telomere length. Aging (Albany NY). 2019;11:5895–923. 31422385 10.18632/aging.102173 PMC6738410

[22] Houseman EA, Accomando WP, Koestler DC, Christensen BC, Marsit CJ, Nelson HH, et al. DNA methylation arrays as surrogate measures of cell mixture distribution. BMC Bioinform. 2012;13:86. 10.1186/1471-2105-13-86 PMC3532182 22568884

[23] Salas LA, Zhang Z, Koestler DC, Butler RA, Hansen HM, Molinaro AM, et al. Enhanced cell deconvolution of peripheral blood using DNA methylation for high-resolution immune profiling. Nat Commun. 2022;13:761. 35140201 10.1038/s41467-021-27864-7 PMC8828780

[24] Higgins-Chen AT, Thrush KL, Wang Y, Minteer CJ, Kuo PL, Wang M, et al. A computational solution for bolstering reliability of epigenetic clocks: Implications for clinical trials and longitudinal tracking. Nat Aging. 2022;2:644–61. 36277076 10.1038/s43587-022-00248-2 PMC9586209

[25] Xu Z, Niu L, Taylor JA. The ENmix DNA methylation analysis pipeline for Illumina BeadChip and comparisons with seven other preprocessing pipelines. Clin Epigenetics. 2021;13:216. 34886879 10.1186/s13148-021-01207-1 PMC8662917

[26] Teschendorff AE, Horvath S. Epigenetic ageing clocks: statistical methods and emerging computational challenges. Nat Rev Genet. 2025;26:350–68. 39806006 10.1038/s41576-024-00807-w

[27] Horvath S, Gurven M, Levine ME, Trumble BC, Kaplan H, Allayee H, et al. An epigenetic clock analysis of race/ethnicity, sex, and coronary heart disease. Genome Biol. 2016;17:171. 27511193 10.1186/s13059-016-1030-0 PMC4980791

[28] Horvath S, Langfelder P, Kwak S, Aaronson J, Rosinski J, Vogt TF, et al. Huntington’s disease accelerates epigenetic aging of human brain and disrupts DNA methylation levels. Aging (Albany NY). 2016;8:1485–512. 27479945 10.18632/aging.101005 PMC4993344

[29] Horvath S, Ritz BR. Increased epigenetic age and granulocyte counts in the blood of Parkinson’s disease patients. Aging (Albany NY). 2015;7:1130–42. 26655927 10.18632/aging.100859 PMC4712337

[30] Levine ME, Lu AT, Bennett DA, Horvath S. Epigenetic age of the pre-frontal cortex is associated with neuritic plaques, amyloid load, and Alzheimer’s disease related cognitive functioning. Aging (Albany NY). 2015;7:1198–211. 26684672 10.18632/aging.100864 PMC4712342

[31] Cardenas A, Ecker S, Fadadu RP, Huen K, Orozco A, McEwen LM, et al. Epigenome-wide association study and epigenetic age acceleration associated with cigarette smoking among Costa Rican adults. Sci Rep. 2022;12:4277. 35277542 10.1038/s41598-022-08160-w PMC8917214

[32] Armanios M. Telomeres and age-related disease: how telomere biology informs clinical paradigms. J Clin Invest. 2013;123:996–1002. 23454763 10.1172/JCI66370 PMC3673231

[33] Yeh JK, Lin MH, Wang CY. Telomeres as therapeutic targets in heart disease. JACC Basic Transl Sci. 2019;4:855–65. 31998853 10.1016/j.jacbts.2019.05.009 PMC6978555

[34] Armanios M. The role of telomeres in human disease. Annu Rev Genomics Hum Genet. 2022;23:363–81. 35609925 10.1146/annurev-genom-010422-091101 PMC10111244

[35] Whittemore K, Vera E, Martinez-Nevado E, Sanpera C, Blasco MA. Telomere shortening rate predicts species life span. Proc Natl Acad Sci U S A. 2019;116:15122–7. 31285335 10.1073/pnas.1902452116 PMC6660761

[36] Bergstedt J, Azzou SAK, Tsuo K, Jaquaniello A, Urrutia A, Rotival M, et al. The immune factors driving DNA methylation variation in human blood. Nat Commun. 2022;13:5895. 36202838 10.1038/s41467-022-33511-6 PMC9537159

[37] Pearce EE, Horvath S, Katta S, Dagnall C, Aubert G, Hicks BD, et al. DNA-methylation-based telomere length estimator: comparisons with measurements from flow FISH and qPCR. Aging (Albany NY). 2021;13:14675–86. 34083495 10.18632/aging.203126 PMC8221337

[38] Terekhova M, Bohacova P, Artyomov MN. Human immune aging. Immunity. 2025;58:2646–69. 41175875 10.1016/j.immuni.2025.10.009

[39] Thin KA, Cross A, Angsuwatcharakon P, Mutirangura A, Puttipanyalears C, Edwards SW. Changes in immune cell subtypes during ageing. Arch Gerontol Geriatr. 2024;122:105376. 38412791 10.1016/j.archger.2024.105376

[40] Liu Z, Liang Q, Ren Y, Guo C, Ge X, Wang L, et al. Immunosenescence: molecular mechanisms and diseases. Signal Transduct Target Ther. 2023;8:200. 37179335 10.1038/s41392-023-01451-2 PMC10182360

[41] Martos SN, Campbell MR, Lozoya OA, Wang X, Bennett BD, Thompson IJB, et al. Single-cell analyses identify dysfunctional CD16(+) CD8 T cells in smokers. Cell Rep Med. 2020;1:100054. 33163982 10.1016/j.xcrm.2020.100054 PMC7644053

[42] Bojesen SE, Timpson N, Relton C, Davey Smith G, Nordestgaard BG. AHRR (cg05575921) hypomethylation marks smoking behaviour, morbidity and mortality. Thorax. 2017;72:646–53. 28100713 10.1136/thoraxjnl-2016-208789 PMC5520281

[43] Dawes K, Andersen A, Reimer R, Mills JA, Hoffman E, Long JD, et al. The relationship of smoking to cg05575921 methylation in blood and saliva DNA samples from several studies. Sci Rep. 2021;11:21627. 34732805 10.1038/s41598-021-01088-7 PMC8566492

[44] Gao X, Zhang Y, Breitling LP, Brenner H. Relationship of tobacco smoking and smoking-related DNA methylation with epigenetic age acceleration. Oncotarget. 2016;7:46878–89. 27276709 10.18632/oncotarget.9795 PMC5216910

