# Supplementary material for: Impact of tobacco smoking on estimated telomere shortening and epigenetic age acceleration among human blood immune cell types
Source: Clin Epigenetics. 2026 Jun 30;18:166. doi: 10.1186/s13148-026-02187-w (PMC13587402; doi:10.1186/s13148-026-02187-w)
Supplement: Supplementary file 1 [file 13148_2026_2187_MOESM1_ESM.docx]

**SUPPLEMENTARY TABLES**

**Table S1.** **Participant demographic summary**

|  | **Nonsmokers (n=74)** | **Smokers (n=69)** | **p-value (test)** |
| --- | --- | --- | --- |
| Age | 39.0 +/- 1.2 (mean+/- SEM*) | 43.2+/- 1.1 (mean+/- SEM) | 0.01 (t-test) |
| BMI | 30.2 +/- 0.9 (mean+/- SEM) | 28.0 +/- 0.6 (mean+/- SEM) | 0.036 (t-test) |
| Race | Black (37), White (33), Other (4) | Black (40), White (25), Other (4) | 0.59 (chisq.test) |
| Sex | Female (41), Male (33) | Female (37), Male (32) | 0.96 (chisq.test) |

*****SEM = Standard Error of the Mean

**Table S2.** **Telomere shortening in blood cell types and whole blood associated with smoking**

|  | | | **Monocyte (CD14+)**  **(n=142)** | | | **Granulocyte (CD15+)**  **(n=138)** | | | **B-cell (CD19+)**  **(n=142)** | | | **Natural Killer (CD56+)**  **(n=135)** | | | **CD4T (CD4+)**  **(n=136)** | | | **CD8T (CD8+)**  **(n=136)** | | | **Matched-Whole Blood**  **(n=101)** | | | **Su et al. [3] Whole Blood (n=253)** | | |
| --- | --- | --- | --- | --- | --- | --- | --- | --- | --- | --- | --- | --- | --- | --- | --- | --- | --- | --- | --- | --- | --- | --- | --- | --- | --- | --- |
| *AHRR* cg05575921 Demethylation *(%)*^a^ | | | - 40% | | | - 40% | | | - 20% | | | - 19% | | | - 7% | | | - 4% | | | - 29% | | | - 26% | | |
| p | | 5.22E-05 | | | 9.02E-04 | | | 7.61E-05 | | | 7.31E-04 | | | 1.79E-02 | | | 2.26E-02 | | | 2.50E-04 | | | 5.83E-13 | | |  |
| p.adj | | 4.39E-06 | | | 1.09E-04 | | | 4.11E-05 | | | 2.73E-03 | | | 4.28E-03 | | | 1.42E-02 | | | 5.17E-05 | | | 2.81E-13 | | |  |
| p.adj.cells6 | | 1.89E-06 | | | 1.10E-04 | | | 4.24E-06 | | | 1.74E-03 | | | 1.80E-02 | | | 4.83E-03 | | | 7.11E-05 | | | 1.43E-12 | | |  |
| p.adj.cells12 | | 9.45E-06 | | | 1.26E-05 | | | 1.19E-04 | | | 4.81E-03 | | | 4.75E-02 | | | 2.39E-02 | | | 4.14E-03 | | | 3.06E-07 | | |  |
| ΔmTL shortening | | -0.14 | | | -0.12 | | | -0.16 | | | -0.21 | | | -0.13 | | | -0.27 | | | -0.14 | | | -0.20 | | |  |
|  |  | | |  | | |  | | |  | | |  | | |  | | |  | | |  | | |  |  |

^a^ The percent methylation change in *AHRR* cg05575921 associated with smoking among each cell type and whole blood

**Table S3.** **Biological age acceleration in blood cell types and whole blood associated with smoking**

|  |  | **Monocyte (CD14+)** | **Granulocyte (CD15+)** | **B-cell (CD19+)** | **Natural Killer (CD56+)** | **CD4T (CD4+)** | **CD8T (CD8+)** | **Matched-Whole Blood** | **Su et al. [3] Whole Blood (n=253)** |
| --- | --- | --- | --- | --- | --- | --- | --- | --- | --- |
|  | *AHRR* cg05575921 Demethylation (%)^a^ | - 40% | - 40% | - 20% | - 19% | - 7% | - 4% | - 29% | - 26% |
| GrimAge2 | p | 1.39E-28 | 1.74E-27 | 9.80E-17 | 2.20E-21 | 2.79E-05 | 4.78E-09 | 8.31E-20 | 9.53E-35 |
|  | p.adj | 8.84E-30 | 6.23E-29 | 1.37E-16 | 5.00E-22 | 3.23E-05 | 2.25E-08 | 3.49E-23 | 4.08E-35 |
|  | p.adj.cells6 | 1.16E-28 | 2.29E-28 | 2.50E-19 | 4.71E-24 | 5.35E-05 | 1.17E-08 | 3.22E-24 | 2.80E-42 |
|  | p.adj.cells12 | 2.01E-27 | 1.05E-28 | 6.45E-20 | 4.76E-20 | 8.57E-07 | 1.20E-07 | 3.58E-19 | 6.14E-34 |
|  | ΔEAA^b^ | 11.44 | 11.74 | 8.67 | 8.15 | 4.30 | 4.71 | 9.15 | 9.24 |
|  |  |  |  |  |  |  |  |  |  |
| FitAge | p | 2.05E-17 | 6.82E-13 | 5.58E-06 | 2.45E-10 | 2.94E-03 | 5.62E-04 | 8.87E-14 | 1.61E-25 |
|  | p.adj | 4.82E-17 | 8.69E-13 | 4.93E-06 | 4.53E-10 | 2.86E-03 | 1.49E-03 | 1.03E-16 | 2.73E-25 |
|  | p.adj.cells6 | 5.14E-17 | 9.04E-16 | 4.89E-07 | 5.03E-14 | 1.32E-02 | 1.33E-03 | 6.04E-14 | 3.19E-26 |
|  | p.adj.cells12 | 2.68E-16 | 5.74E-16 | 1.72E-05 | 4.78E-11 | 7.54E-03 | 5.44E-03 | 6.42E-11 | 3.00E-19 |
|  | ΔEAA | 7.68 | 7.02 | 6.13 | 6.96 | 2.71 | 3.90 | 7.01 | 6.40 |
|  |  |  |  |  |  |  |  |  |  |
| PhenoAge | p | 9.58E-03 | 1.31E-04 | 9.10E-03 | 6.83E-04 | *1.12E-01* | 4.51E-03 | 9.00E-03 | 4.23E-06 |
|  | p.adj | 3.64E-03 | 8.24E-05 | 2.20E-02 | 3.94E-03 | *6.74E-02* | 8.68E-03 | 5.70E-03 | 3.20E-05 |
|  | p.adj.cells6 | 2.14E-03 | 7.16E-04 | *7.45E-02* | 8.11E-03 | *1.94E-01* | 1.12E-03 | *7.24E-02* | 5.48E-05 |
|  | p.adj.cells12 | 2.88E-03 | 8.80E-04 | *5.20E-01* | 3.85E-03 | *2.91E-01* | 4.97E-02 | *4.92E-01* | *5.59E-02* |
|  | ΔEAA | 2.86 | 5.05 | 3.81 | 4.73 | 2.48 | 6.54 | 2.78 | 3.90 |
|  |  |  |  |  |  |  |  |  |  |
| Hannum | p | *1.35E-01* | *6.77E-01* | *1.47E-01* | 1.17E-02 | *9.50E-01* | *1.56E-01* | 2.42E-02 | *1.11E-01* |
|  | p.adj | *8.28E-02* | *4.21E-01* | *2.96E-01* | 3.98E-02 | *7.99E-01* | *1.89E-01* | 2.32E-02 | *3.05E-01* |
|  | p.adj.cells6 | *8.55E-02* | *3.42E-01* | *4.60E-01* | 2.27E-02 | *9.36E-01* | *1.65E-01* | 1.13E-02 | *4.19E-01* |
|  | p.adj.cells12 | *1.38E-01* | *5.27E-01* | *9.36E-01* | *8.59E-02* | *3.74E-01* | *5.55E-01* | *1.46E-01* | *1.50E-01* |
|  | ΔEAA | 1.10 | 0.30 | 1.64 | 3.25 | 0.06 | 2.60 | 1.53 | 0.98 |

| Skin_Blood | p | *3.43E-01* | *7.55E-01* | 2.34E-02 | *1.50E-01* | *5.84E-02* | 1.63E-02 | *6.15E-02* | *4.45E-01* |  |
| --- | --- | --- | --- | --- | --- | --- | --- | --- | --- | --- |
|  | p.adj | *3.16E-01* | *8.02E-01* | 4.51E-02 | *3.28E-01* | *5.49E-02* | *5.32E-02* | 9.71E-03 | *6.10E-01* |  |
|  | p.adj.cells6 | *3.17E-01* | *7.40E-01* | *4.01E-01* | *2.43E-01* | *5.93E-02* | 3.70E-02 | 1.09E-02 | *7.06E-01* |  |
|  | p.adj.cells12 | *2.02E-01* | *8.10E-01* | *8.80E-01* | *2.59E-01* | *1.14E-01* | *4.36E-01* | 4.77E-02 | *5.65E-01* |  |
|  | ΔEAA | 0.68 | -0.24 | 1.97 | 1.52 | 1.38 | 2.45 | 1.36 | 0.42 |  |

| Horvath | p | *9.19E-01* | *7.60E-01* | *1.16E-01* | *2.74E-01* | *2.55E-01* | *3.11E-01* | *9.50E-01* | *5.82E-02* |
| --- | --- | --- | --- | --- | --- | --- | --- | --- | --- |
|  | p.adj | *8.31E-01* | *9.88E-01* | *1.55E-01* | *5.09E-01* | *3.82E-01* | *5.86E-01* | *8.31E-01* | *2.33E-01* |
|  | p.adj.cells6 | *6.88E-01* | *7.43E-01* | *1.45E-01* | *3.24E-01* | *6.30E-01* | *6.06E-01* | *5.74E-01* | *4.76E-01* |
|  | p.adj.cells12 | *5.78E-01* | *9.04E-01* | *1.53E-01* | *1.83E-01* | *6.71E-01* | *5.43E-01* | *6.62E-01* | *8.34E-01* |
|  | ΔEAA | 0.09 | -0.29 | 1.32 | 1.70 | 0.93 | 1.23 | -0.06 | 1.19 |

^a^ The percent methylation change in *AHRR* cg05575921 associated with smoking among each cell type and whole blood

^b^ΔEAA is the difference in epigenetic age acceleration among smokers and nonsmokers (SM – NS)

**Table S4.** **Changes in DunedinPACE due to smoking among cell types and whole blood**

|  | | **Monocyte (CD14+)** | | **Granulocyte (CD15+)** | | **B-cell (CD19+)** | | | **Natural Killer (CD56+)** | | | **CD4T (CD4+)** | | | **CD8T (CD8+)** | | | **Matched-Whole Blood** | | | **Su et al. [3] Whole Blood (n=253)** | |  |
| --- | --- | --- | --- | --- | --- | --- | --- | --- | --- | --- | --- | --- | --- | --- | --- | --- | --- | --- | --- | --- | --- | --- | --- |
| *AHRR* cg05575921 Demethylation (*%)* | | - 40% | | - 40% | | - 20% | | | - 19% | | | - 7% | | | - 4% | | | - 29% | | | - 26% | |  |
| p | | 6.67E-12 | | 1.65E-12 | | | 1.19E-04 | | | 2.75E-09 | | | 1.17E-05 | | | 2.05E-04 | | | 6.26E-09 | | | 3.72E-19 | |
| p.adj | | 8.11E-12 | | 5.67E-14 | | | 1.77E-04 | | | 2.42E-08 | | | 2.79E-04 | | | 3.26E-03 | | | 7.19E-11 | | | 2.72E-15 | |
| p.adj.cells6 | | 1.51E-12 | | 5.60E-13 | | | 6.42E-09 | | | 4.01E-08 | | | 6.06E-05 | | | 8.36E-06 | | | 2.18E-09 | | | 1.04E-15 | |
| p.adj.cells12 | | 1.43E-11 | | 7.72E-13 | | | 2.48E-08 | | | 1.78E-07 | | | 2.05E-03 | | | 2.30E-03 | | | 2.32E-07 | | | 3.42E-12 | |
| ΔPACE* | | 0.14 | | 0.17 | | | 0.08 | | | 0.13 | | | 0.09 | | | 0.08 | | | 0.14 | | | 0.18 | |
|  |  | |  | |  | | |  | | |  | | |  | | |  | | |  |  |  |  |

* ΔPACE is the difference in DunedinPACE among smokers and nonsmokers (SM – NS)

**Table S5.** **Principal component version of telomere shortening in blood cell types and whole blood associated with smoking status**

|  | | | **Monocyte (CD14+)** | | | **Granulocyte (CD15+)** | | | **B-cell (CD19+)** | | | **Natural Killer (CD56+)** | | | **CD4T (CD4+)** | | | **CD8T (CD8+)** | | | **Matched Whole blood** | | | | | **Su et al. [3] Whole blood (n=253)** | | | |
| --- | --- | --- | --- | --- | --- | --- | --- | --- | --- | --- | --- | --- | --- | --- | --- | --- | --- | --- | --- | --- | --- | --- | --- | --- | --- | --- | --- | --- | --- |
| *AHRR* cg05575921 *Demethylation (%)* | | | - 40% | | | - 40% | | | - 20% | | | - 19% | | | - 7% | | | - 4% | | | - 29% | | | | | - 26% | | | |
| p | | 1.17E-03 | | | 1.25E-03 | | | 4.91E-03 | | | 1.48E-03 | | | 5.01E-02 | | | 1.53E-02 | | | | | 1.80E-05 | | 1.37E-12 | | | |  |  |
| p.adj | | 5.46E-06 | | | 9.73E-06 | | | 2.98E-03 | | | 6.37E-03 | | | 9.32E-02 | | | 5.94E-02 | | | | | 5.45E-06 | | 3.31E-11 | | | |  |  |
| p.adj.cells6 | | 2.69E-06 | | | 3.72E-06 | | | 5.85E-04 | | | 2.86E-03 | | | *4.77E-01* | | | 2.27E-02 | | | | | 6.85E-06 | | 5.75E-11 | | | |  |  |
| p.adj.cells12 | | 1.75E-05 | | | 1.07E-06 | | | 3.38E-02 | | | 4.29E-03 | | | *3.76E-01* | | | *2.05E-01* | | | | | 5.35E-04 | | 3.34E-05 | | | |  |  |
| Δ DNAmTL | | -0.07 | | | -0.07 | | | -0.12 | | | -0.18 | | | -0.09 | | | -0.19 | | | -0.12 | | | | | -0.15 | | | |  |
|  |  | | |  | | |  | | |  | | |  | | |  | | |  | | | |  | | | |  |  |  |
|  |  | | |  | | |  | | |  | | |  | | |  | | |  | | | |  | | | |  |  |  |

**Table S6.** **Principal component version of epigenetic age acceleration in blood cell types associated with smoking status**

|  |  | **Monocyte (CD14+)** | **Granulocyte (CD15+)** | **B-cell (CD19+)** | **Natural Killer (CD56+)** | **CD4T (CD4+)** | **CD8T (CD8+)** | **Matched Whole Blood** | **Su et al Whole Blood (n=253)** |
| --- | --- | --- | --- | --- | --- | --- | --- | --- | --- |
|  | *AHRR* cg05575921 *Demethylation (%)* | - 40% | - 40% | - 20% | - 19% | - 7% | - 4% | - 29% | - 26% |
| PCGrimAge | p | 1.62E-30 | 1.73E-27 | 8.16E-15 | 4.06E-15 | 4.29E-06 | 2.76E-10 | 1.48E-21 | 4.99E-30 |
|  | p.adj | 1.90E-31 | 3.63E-28 | 4.21E-16 | 8.49E-16 | 8.34E-07 | 7.22E-10 | 1.93E-23 | 3.38E-29 |
|  | p.adj.cells6 | 6.79E-30 | 1.21E-28 | 7.12E-20 | 8.75E-23 | 1.01E-07 | 5.40E-13 | 4.33E-21 | 1.75E-35 |
|  | p.adj.cells12 | 5.75E-28 | 2.70E-31 | 1.10E-19 | 3.00E-18 | 2.00E-11 | 1.42E-10 | 7.20E-18 | 2.80E-28 |
|  | ΔEAA_SM | 7.41 | 7.42 | 5.18 | 5.26 | 2.65 | 3.80 | 6.83 | 7.02 |
|  |  |  |  |  |  |  |  |  |  |
| PCPhenoAge | p | *3.79E-01* | *1.24E-01* | *6.67E-02* | 3.63E-03 | *1.37E-01* | 6.09E-03 | 3.70E-02 | 8.84E-04 |
|  | p.adj | *9.53E-02* | 1.89E-02 | 3.63E-02 | 3.75E-03 | *1.21E-01* | 2.93E-02 | 4.20E-03 | 3.00E-03 |
|  | p.adj.cells6 | *1.02E-01* | 3.28E-02 | *7.20E-02* | 7.94E-03 | *4.08E-01* | 6.59E-03 | 1.75E-02 | 1.54E-03 |
|  | p.adj.cells12 | *9.11E-02* | *5.83E-02* | *8.21E-01* | 1.52E-02 | *4.35E-01* | *3.82E-01* | *1.51E-01* | *7.86E-01* |
|  | ΔEAA_SM | 0.75 | 1.38 | 3.24 | 3.97 | 2.00 | 4.99 | 2.31 | 3.22 |
|  |  |  |  |  |  |  |  |  |  |
| PCHannum | p | *9.85E-01* | *7.58E-01* | *4.98E-01* | *5.36E-02* | *2.71E-01* | *5.52E-02* | 2.25E-03 | 3.55E-02 |
|  | p.adj | *4.88E-01* | *6.16E-01* | *4.99E-01* | *1.61E-01* | *2.43E-01* | *1.81E-01* | 6.25E-04 | *7.77E-02* |
|  | p.adj.cells6 | *4.08E-01* | *1.69E-01* | *6.58E-01* | *1.28E-01* | *9.35E-01* | *2.39E-01* | 2.47E-02 | *1.15E-01* |
|  | p.adj.cells12 | *1.96E-01* | *2.80E-01* | *3.86E-01* | *1.71E-01* | *8.89E-01* | *9.17E-01* | *5.43E-02* | *3.97E-01* |
|  | ΔEAA_SM | -0.02 | -0.26 | 0.96 | 2.37 | 1.19 | 3.16 | 2.66 | 1.35 |

| PCHorvath2  (PCSkin_Blood) | p | *6.72E-01* | *6.48E-01* | *1.16E-01* | 1.47E-02 | 2.79E-02 | 4.26E-02 | *5.56E-02* | 1.35E-04 |  |
| --- | --- | --- | --- | --- | --- | --- | --- | --- | --- | --- |
|  | p.adj | *7.71E-01* | *6.94E-01* | *1.40E-01* | *6.90E-02* | 4.03E-02 | *1.45E-01* | 2.38E-02 | 2.61E-04 |  |
|  | p.adj.cells6 | *7.32E-01* | *3.01E-01* | *3.29E-01* | *7.38E-02* | *2.46E-01* | *1.48E-01* | *9.02E-02* | 1.22E-03 |  |
|  | p.adj.cells12 | *3.17E-01* | *6.19E-01* | *8.02E-01* | *7.49E-02* | *4.23E-01* | *8.12E-01* | *1.84E-01* | *1.34E-01* |  |
|  | ΔEAA_SM | -0.40 | -0.47 | 2.62 | 3.87 | 2.16 | 3.51 | 1.85 | 2.59 |  |

| PCHorvath | p | *8.37E-01* | *8.44E-01* | *3.81E-01* | 2.73E-02 | *6.99E-02* | 2.57E-02 | 2.87E-02 | 2.67E-03 |
| --- | --- | --- | --- | --- | --- | --- | --- | --- | --- |
|  | p.adj | *5.93E-01* | *4.64E-01* | *2.88E-01* | *1.14E-01* | *7.30E-02* | *8.84E-02* | 2.33E-02 | 6.27E-03 |
|  | p.adj.cells6 | *4.40E-01* | *2.35E-01* | *4.63E-01* | *1.27E-01* | *3.42E-01* | *8.66E-02* | *1.67E-01* | 3.57E-02 |
|  | p.adj.cells12 | *1.74E-01* | *3.98E-01* | *9.48E-01* | *1.11E-01* | *4.55E-01* | *7.29E-01* | *1.05E-01* | *5.41E-01* |
|  | ΔEAA | 0.19 | 0.19 | 0.93 | 2.66 | 1.52 | 2.80 | 1.93 | 1.85 |

**Table S7.** **Association of stemTOC in isolated blood cell types and whole blood with smoking status**

|  | | **Monocyte (CD14+)** | | **Granulocyte (CD15+)** | | **B-cell (CD19+)** | | | **Natural Killer (CD56+)** | | | **CD4T (CD4+)** | | | **CD8T (CD8+)** | | | **Matched-Whole Blood** | | | **Su et al. [3] Whole Blood (n=253)** | |  |
| --- | --- | --- | --- | --- | --- | --- | --- | --- | --- | --- | --- | --- | --- | --- | --- | --- | --- | --- | --- | --- | --- | --- | --- |
| *AHRR* cg05575921 Demethylation (*%)* | | - 40% | | - 40% | | - 20% | | | - 19% | | | - 7% | | | - 4% | | | - 29% | | | - 26% | |  |
| p | | *4.89E-01* | | *5.38E-02* | | | 5.82E-03 | | | 6.37E-03 | | | *1.91E-01* | | | 3.09E-02 | | | 2.74E-02 | | | 9.38E-04 | |
| p.adj | | *8.51E-01* | | *1.18E-01* | | | *5.28E-02* | | | *6.95E-02* | | | *6.69E-01* | | | *2.90E-01* | | | *7.77E-02* | | | 2.53E-02 | |
| p.adj.cells6 | | *6.48E-01* | | *4.11E-01* | | | *1.51E-01* | | | *1.29E-01* | | | *5.87E-01* | | | *8.12E-02* | | | 1.87E-02 | | | 4.64E-03 | |
| p.adj.cells12 | | *7.75E-01* | | *8.71E-02* | | | *6.74E-01* | | | *3.73E-01* | | | *4.76E-01* | | | *5.20E-01* | | | 4.89E-02 | | | *8.61E-01* | |
| ΔstemTOC | | 0.002 | | 0.007 | | | 0.031 | | | 0.044 | | | 0.012 | | | 0.030 | | | 0.017 | | | 0.015 | |
|  |  | |  | |  | | |  | | |  | | |  | | |  | | |  |  |  |  |

* ΔstemTOC is the difference in average stemTOC values among smokers and nonsmokers (SM – NS)
